# Effective use of polymyxin B hemoperfusion in septic shock complicated by urosepsis

**DOI:** 10.4103/0971-4065.78077

**Published:** 2011

**Authors:** P. Malleshappa, R. Ranganath, A. P. Chaudhari, P. Singhai, M. Aghariya, A. B. Shah

**Affiliations:** Department of Nephrology, Lilavati Hospital and Research Centre, Mumbai, India

**Keywords:** Septic shock, Gram-negative infection, urosepsis, PMX-DHP

## Abstract

Direct hemoperfusion using polymyxin B–immobilized fiber (PMX-DHP) is an established treatment method for septic shock caused by Gram-negative infections. Here we report one instance in which PMX-DHP therapy has been used successfully in a patient with septic shock from urosepsis. After antibiotic therapy, direct hemoperfusion using polymyxin B helped in cardiovascular stability. The patient recovered from the shock within a few days after treatment with polymyxin-B hemoperfusion. As far as we are aware, this is the first reported case of effective treatment of urosepsis complicated by septic shock using PMX-DHP therapy in India.

## Introduction

Shock is a life-threatening complication of serious Gram-negative bacteremia, and the genitourinary tract is an important source of bacteremia. Sepsis involves a complex interaction between bacterial toxins and the host immune system. Endotoxin is considered one of the principal biological substances implicated in the genesis of septic shock.[1] Nevertheless, anti-endotoxin drug therapies failed to demonstrate a consistent clinical benefit. This lack of clinical success has shifted interest to extracorporeal therapies to reduce circulating levels of endotoxin. Direct hemoperfusion using polymyxin B–immobilized fiber is a blood purification therapy, which was developed to adsorb blood endotoxins in serious infectious disease states resulting from Gram-negative bacterial infections. We report the effect of direct hemoperfusion using polymyxin B–immobilized fiber in a patient with septic shock from urosepsis.

## Case Report

A 72-year-old man presented with high-grade fever with chills and dysuria for last 10 days, decreased urine output, and worsening breathlessness since 1 day. He denied any history of hematuria, pain in abdomen, skin rashes, or joint pains. He was a diabetic and hypertensive on regular treatment since 5 years. Physical examination revealed a middle aged man in acute respiratory distress with a respiratory rate of 32 beats/min. His blood pressure was 150/90 mmHg and his temperature was 100° F. The jugular venous pressure was elevated, he had bilateral pitting edema of the legs, and sinus tachycardia was present (108/min with an S3 gallop). Auscultation of the lungs revealed pulmonary congestion.

His laboratory investigations were as follows: hemoglobin 9.1 g/dL, total leukocyte count 23,200/cmm, platelet count 592,000/cmm, BUN 65 mg/dL, creatinine 1.8 mg/dL, sodium 139 mEq/L, potassium 4.3 mEq/L, chloride 95.3 mEq/L, bicarbonate 13.8 mEq/L, total bilirubin 1.3 mg/dL, direct bilirubin 0.3 mg/dL, SGOT 32 IU/L, SGPT 25 IU/L, ALP 210 IU/L, total proteins 7.7 g/dL, albumin 3.1 g/dL, C-reactive protein 142, amylase 71, lipase 82, procalcitonin 1.4. His urine microscopy revealed trace proteins with entire field full of pus cells. A chest radiograph confirmed pulmonary edema. His abdominal sonography was unremarkable except for moderate ascites. CT-abdomen and pelvis revealed acute pyelonephritis with perinephric fat stranding.

The patient was transferred to the intensive care unit. Diuretic therapy was intensified (frusemide infusion), he was put on noninvasive ventilation, and broad-spectrum intravenous antibiotics were administered (meropenem, teicoplanin, aztreonem). His urine culture came positive for *Escherichia coli*, whereas blood culture was sterile. He continued to have high-grade fever and went into septic shock, with the acute physiology and chronic health evaluation (APACHE) II score of 21 and the sepsis-related organ failure assessment (SOFA) score of 9. He was intubated, put on ionotropic support, and antibiotics were changed according to sensitivity reports (tigecycline, colistin, vancomycin, clindamycin). His blood pressure stabilized at 100/50 mmHg on dopamine infusion at 20 μg/kg/min, noradrenaline at 15 μg/kg/min, adrenaline at 0.1 μg/kg/min, and vasopressin at 0.02 U/min. He was initiated on sustained low-efficiency hemodialysis (SLED), following anuria, worsening azotemia, and severe metabolic acidosis. In view of on-going sepsis and unstable hemodynamics, decision was taken to initiate him on direct hemoperfusion using PMX-DHP.

After priming the cartridge and blood lines, 2 hrs of direct hemoperfusion was performed using a blood flow rate of 100 mL/min and heparin anticoagulation. Twelve hours after the initiation of PMX-DHP therapy, vasopressin and adrenaline infusions were tapered, and there was a noticeable improvement in his urine output. 24 hr after initiation of PMX-DHP, vasopressin and adrenaline infusions were stopped. After another 24 hours, the dopamine and noradrenaline doses were decreased, and the urine output increased to approximately 0.7 mL/kg/hr. Under these conditions, the heart rate was maintained at 110 bpm; blood pressure 135/60 mmHg with a mean arterial pressure (MAP) of 85 mmHg [Table 1]. The SOFA score improved from 9 to 6, and the APACHE II score improved from 21 to 11 (since the patient was under sedation, the score for the central nervous system was excluded). He required four more days of SLED, and his urine output increased to greater than 1 mL/kg/hr and he was taken off dialysis. He was discharged 60 days after the hemoperfusion therapy, with a stable creatinine of 2 mg/dL and on empirical antitubercular therapy.

**Table 1:** Evolution of clinical status during and after hemoperfusion

	Pretreatment with PMX-DHP	12 hr post PMX—DHP	24 hr	48 hr
Heart rate (beats per min)	125	120	115	110
Systolic BP (mmHg)	100	125	130	135
Diastolic BP (mmHg)	50	55	60	60
Mean arterial pressure (mmHg)	67	78	83	85
Dopamine (μg/kg/min)	20	20	10	8
Noradrenaline (μg/kg/min)	15	15	8	4
Adrenaline (μg/kg/min)	0.1	0.05	Nil	Nil
Vasopressin (U/min)	0.02	0.01	Nil	Nil
Urine output (mL/kg/hr)	Nil	0.3	0.5	0.7

## Discussion

Septic shock is a life-threatening disorder, and it has been hypothesized that endotoxin, a cell-wall component of Gram-negative bacteria, is a central initiator of Gram-negative septic shock. Current therapy is not effective once the cascade of septic shock is initiated. Polymyxin B is a well-known antibiotic for removing endotoxin, but its use is limited only to oral or local administration because of its strong neurotoxicity and nephrotoxicity.[2] Therefore, it cannot be used for the treatment of endotoxemia.

Polymyxin B has been bound and immobilized to polystyrene fibers in a medical device for hemoperfusion. This device can effectively bind endotoxin both *in vitro* and *in vivo* and could potentially interrupt the biological cascade of sepsis.[3] In a systematic review, direct hemoperfusion with the polymyxin B device appeared to have favorable effects on MAP, vasopressor use, and mortality.[4] In a small European pilot study, polymyxin B was shown to improve cardiac and renal dysfunction due to sepsis or septic shock but had no effect on mortality.[5] More recently, another small randomized study demonstrated favorable effects of polymyxin B on renal tubular cell structure and function.[6] In the EUPHAS randomized controlled trial, the 28-day crude mortality was 32% (11/34 patients) in the polymyxin B hemoperfusion group and 53% (16/30 patients) in the conventional therapy group.[7] The statistically significant difference in mortality rates reported in this study was based on the analysis of a hazard ratio (HR) generated by a Cox proportional hazards regression survival model. With univariable analysis, only treatment group and SOFA score were independently associated with mortality. After adjusting for SOFA score, the polymixin B hemoperfusion group had a significant reduction in 28-day mortality (adjusted HR, 0.36; 95% confidence interval (CI), 0.16-0.80; *P*=0.01). In a further analysis of hospital mortality, 20 of 30 patients (67%) died in the conventional therapy group compared with 14 of 34 patients (41%) in the polymixin B hemoperfusion group. After adjusting for SOFA score, the polymixin B hemoperfusion group had a significant reduction in hospital mortality rate (adjusted HR, 0.43; 95% CI, 0.21-0.90; *P*=0.026 [Pocock value for 1 interim analysis, *P*<0.029]). There was no significant change in the proportion of patients receiving renal replacement therapy (RRT) at 72 hr in either group. Mean mechanical ventilation free days in the hospital were similar between the two groups (polymixin B hemoperfusion: 21.4 days; 95% CI, 15.4–27.3 days; vs. conventional therapy: 17.0 days; 95% CI, 8.5–25.3 days; *P*=0.47). Likewise, there was no significant difference in mean length of stay in the ICU (20.3 days; 95% CI, 15.0–25.5 days; vs. 18.3 days; 95% CI, 8.8–27.8 days; *P*=0.72) or in the hospital (37.2 days; 95% CI, 29.6–44.8 days; vs. 32.0 days; 95% CI, 18.0–46.0 days; *P*=0.53). They concluded that polymyxin B hemoperfusion added to the conventional therapy significantly improved hemodynamics and organ dysfunction and reduced 28-day mortality in a targeted population with severe sepsis and/or septic shock from intra-abdominal Gram-negative infections.[7]

In our patient, the general intensive treatment proved ineffective during the first few hours of sepsis, so we tested the use of this newly developed therapeutic method to adsorb endotoxin, and it proved to be efficacious. The systemic vascular resistance, blood pressure improved with PMX-DHP and the patient recovered from the shock; thereafter, the vasopressor doses were reduced. We consider that PMX-DHP is useful for improving the hemodynamic status, and a desirable outcome can be obtained if it is performed at an early stage.

## References

[1] Marshall J, Foster D, Vincent J (2004). Diagnostic and prognostic implications of endotoxemia in critical illness: results of the MEDIC study. J Infect Dis.

[2] Aoki H, Kodama M, Tani T, Hanasawa K (1994). Treatment of sepsis by extracorporeal elimination of endotoxin using polymyxin B-immobilized fiber. Am J Surg.

[3] Shoji H (2003). Extracorporeal endotoxin removal for the treatment of sepsis: endotoxin adsorption cartridge (Toraymyxin). Ther Apher Dial.

[4] Cruz DN, Perazella MA, Bellomo R (2007). Effectiveness of polymyxin B-immobilized fiber column in sepsis: a systematic review. Crit Care.

[5] Vincent JL, Laterre PF, Cohen J (2005). A pilot controlled study of a polymyxin B-immobilized hemoperfusion cartridge in patients with severe sepsis secondary to intra-abdominal infection. Shock.

[6] Cantaluppi V, Assenzio B, Pasero D (2008). Polymyxin-B hemoperfusion inactivates circulating pro-apoptotic factors. Intensive Care Med.

[7] Dinna NC, Massimo A, Roberto F (2009). Early use of polymyxin B hemoperfusion in abdominal septic shock: the EUPHAS randomized controlled trial. JAMA.

